# Ocular Manifestations of Human Immunodeficiency Virus Infection in the Combination Antiretroviral Therapy Era

**DOI:** 10.3390/pathogens12121417

**Published:** 2023-12-04

**Authors:** Mingming Yang, Koju Kamoi, Yuan Zong, Jing Zhang, Yaru Zou, Kyoko Ohno-Matsui

**Affiliations:** Department of Ophthalmology & Visual Science, Graduate School of Medical and Dental Sciences, Tokyo Medical and Dental University, Tokyo 113-8510, Japan; yangmm12.oph@tmd.ac.jp (M.Y.); zongyuan666.oph@tmd.ac.jp (Y.Z.); zhangjing.oph@tmd.ac.jp (J.Z.); alicezouyaru519@gmail.com (Y.Z.); k.ohno.oph@tmd.ac.jp (K.O.-M.)

**Keywords:** human immunodeficiency virus, ocular manifestations, combination antiretroviral therapy, uveitis, cytomegalovirus retinitis, CD4+

## Abstract

Since the introduction of combination antiretroviral therapy (cART) in Japan in 2008, the spectrum of ocular manifestations in patients with human immunodeficiency virus (HIV) has changed. This study, conducted at Tokyo Medical and Dental University Hospital between January 2012 and August 2023, aimed to understand the epidemiology and clinical features of ocular manifestations in patients with HIV during the cART era. Of the 218 patients diagnosed with HIV, 23 (10.55%) exhibited ocular manifestations; all were male, aged 32–73. The most prevalent ocular complication was uveitis (60.67%). Notably, the prevalence of uveitis in this cART era has surged compared to earlier Japanese studies. Our data also suggest a potential direct link between uveitis and HIV, particularly in patients who have not yet undergone cART. However, cytomegalovirus retinitis, another prevalent ocular disease in our study, appeared more strongly associated with patients who commenced cART. Neither ocular condition was significantly correlated with CD4+ T-cell count. Importantly, our observed ocular manifestation prevalence (10.55%) was lower than that in previous studies, emphasizing the potential influence of cART and national healthcare support. These findings provide unique insights into the evolution of ocular manifestations in patients with HIV in Japan amidst cART availability.

## 1. Introduction

Since the first human immunodeficiency virus (HIV)-infected individual was reported in 1985, HIV has been a public health concern in Japan for many years [1]. In 2013, the number of newly reported HIV cases in Japan peaked and then decreased slightly. Between 1985 and 2019, the cumulative number of notified cases in Japan was 31,384 for “HIV” (19,216 males; 2523 females) and “acquired immunodeficiency syndrome (AIDS)” (8793 males; 853 females) [2]. In Japan, combination antiretroviral therapy (cART) became available in 2008 following its introduction in 1996. The introduction of cART has significantly extended the lifetime of patients with HIV infection. Approximately 69% of people living with HIV receive cART [3]. Despite advancements in HIV treatment and outcomes, patients still face difficulties due to co-infections and complications. Ocular manifestations are among the most common and important issues in patients with HIV infection, and the spectrum of ocular manifestations has changed since the pre-cART era. Reports from other countries indicate that ocular manifestations in patients with HIV infection have a prevalence ranging from 37.7 to 75% [4]. Studies from developing countries have shown that approximately 5–25% of patients with HIV may develop blindness during their lifetime [5]. The spectrum and epidemiological characteristics of ocular diseases in patients with HIV vary by country. Currently, most reports about ocular manifestations in patients with HIV are from high-HIV-burden areas, such as South Africa and India, and only a few have been reported in Japan. In this study, we investigated the epidemiology and clinical features of ocular manifestations in 23 consecutive patients with HIV infection at Tokyo Tertiary Hospital in the era of cART.

## 2. Materials and Methods

We performed a retrospective chart review of patients who visited the Tokyo Medical and Dental University Hospital between January 2012 and August 2023. The Tokyo Medical and Dental University Hospital is a tertiary hospital located in Tokyo, Japan. HIV seropositivity was established using HIV-1 and HIV-2 antibody tests. Western blot analysis was performed using the positive assays for confirmation [6]. The patients underwent ophthalmic examinations, including best-corrected visual acuity (BCVA), intraocular pressure, slit-lamp biomicroscopy, and dilated fundus examination. The suspected cases were examined using relevant ophthalmological and systemic investigations. The absolute CD4+ T-cell count was determined in most patients (18 patients).

Cytomegalovirus (CMV) retinitis is clinically diagnosed as discrete foci of retinal necrosis with irregular, opacified borders that are granular in character and a variable number of adjacent, small, dot-like, white satellite lesions. Cases of uveitis were classified according to the International Uveitis Study Group classification [7], and unclassified uveitis was diagnosed after extensive examinations for differential diagnosis and uncategorized into specific clinical entities. Optic disc pit maculopathy was diagnosed based on the presence of unilateral oval-shaped excavations in the temporal region of the optic disc accompanied by maculopathy. Other ocular complications, such as retinal detachment (RD), normal-tension glaucoma (NTG), central retinal vein occlusion (CRVO), and proliferative vitreoretinopathy (PVR), were diagnosed by certified ophthalmologists using standardized descriptions and characteristic clinical features.

The data were evaluated and statistically analyzed using the SPSS and Prism software. Statistical significance tests of the number of eyes within different situations were performed using Fisher’s exact test or the chi-square test according to frequencies predicted by the null hypothesis for bivariable analysis. Statistical significance was set at *p* <0.05.

All protocols used in this study were reviewed and approved by the Institutional Ethics Committee of Tokyo Medical and Dental University (IRB No. M2023-126).

## 3. Results

**Figure 1:**
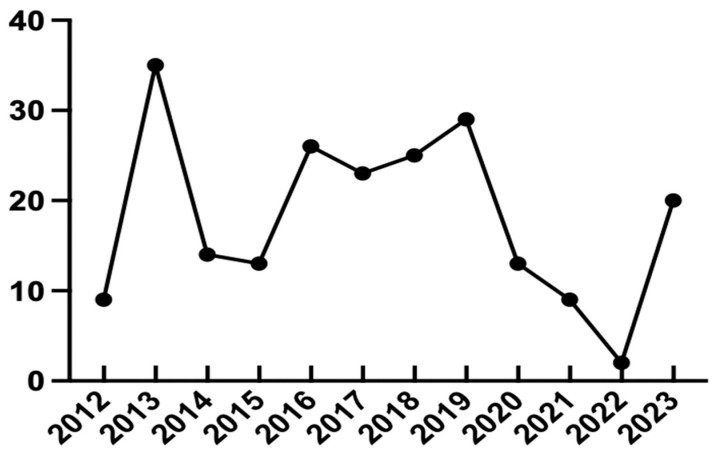
Number of patients diagnosed with HIV each year from 2012 to 2023.

Between January 2012 and August 2023, 218 patients were diagnosed with HIV infection at the Tokyo Medical and Dental University Hospital. Newly diagnosed cases peaked in 2013 (Figure 1), consistent with the 2019 Japanese national survey [2]. Twenty-three (36 eyes) (10.55%) patients presented with ocular manifestations and underwent ophthalmic examination. Among them, 21 (91.30%) patients were diagnosed with HIV and referred to our outpatient unit for ophthalmic examination. The remaining two patients were initially ophthalmic outpatients with ocular manifestations and were suspected to have HIV infection, which was confirmed in later tests. All 23 patients were male, with ages ranging from 32 to 73 and a mean age of 53.13 ± 12.94 years. Among the 23 patients, the majority were in their fifties (*n* = 8, 34.8%), followed by thirties, forties, and sixties (each group *n* = 4, 17.4%). The absolute CD4+ T-cell count was accessible for 18 patients, with a mean count of 416.4 ± 475.7 cells/μL. The demographic and laboratory characteristics of all 23 patients are presented in Table 1.

Among the 23 patients, the most common ophthalmic manifestation related to HIV was uveitis (*n* = 14, 60.67%). Uveitis included anterior uveitis (*n* = 2, including one with varicella-zoster virus (VZV) iritis), posterior/pan uveitis (*n* = 6, including three with syphilitic uveitis), and CMV retinitis (*n* = 6). Among the eight patients with uveitis, four had bilateral involvement, and four had unilateral involvement. Among the six patients with CMV retinitis, only one had bilateral involvement. Cataracts were also common in patients with HIV infection, with nine (39.13%) patients having cataracts in 14 eyes. RD was observed in four patients (*n* = 4, 17.39%). Among these four patients, two had high myopia, and one had CMV retinitis. One patient had herpes zoster ophthalmicus (*n* = 1, 4.35%), and retinal hemorrhage was found in one patient (*n* = 1, 4.35%). The complete ocular manifestations in the 23 patients are presented in Table 2.

Out of 18 patients, 6 (33.33%) had a CD4+ T-cell count below 100 cells/μL. CMV retinitis and uveitis were the most prevalent ocular manifestations in both patient groups, regardless of their CD4+ T-cell counts (above or below 100), indicating a similarity in the spectrum of ophthalmic manifestations between the two groups (Table 3). The mean CD4+ T-cell count for patients with uveitis and CMV retinitis was 433.2 ± 535.6 cells/μL and 585.0 ± 734.3 cells/μL, respectively. CD4+ T-cell counts showed no significant difference between patients with CMV retinitis and those with uveitis (*p* = 0.35).

Twelve (52.2%) patients had received or were receiving cART when they were first examined in our outpatient department. Among these patients, CMV retinitis was the most common ocular manifestation (*n* = 5). However, in patients who had not yet received cART when they first came, unclassified uveitis was the most common manifestation (*n* = 3). More patients with CMV retinitis had either already received or were receiving cART (*p* = 0.0217). Conversely, patients with uveitis were more likely not to have started cART when they had ocular manifestations. However, no statistically significant difference was observed in the incidence of uveitis between the two groups. Most patients with posterior manifestations, including RD (*n* = 4), retinal hemorrhage (*n* = 1), PVR (*n* = 1), and optic disc pit maculopathy, had already received or were receiving cART. Detailed information is provided in Table 4.

## 4. Discussion

Our research aimed to understand the ocular manifestations in patients with HIV infection over the last decade, building upon a foundational study conducted in Japan in 2008 [8]. Notably, the patients in the 2008 study predominantly belonged to the pre-cART era, as cART became available in Japan in the same year [1]. This finding highlights the significance of our study in providing updated insights into ocular manifestations of HIV in the context of cART availability.

The trend of HIV diagnoses from Tokyo Medical and Dental University Hospital, peaking in 2013, correlates with the national survey data from Japan in 2019. This aligns with our findings, which also observed that 2013 was the year with the highest number of HIV diagnoses.

The majority of our study participants were middle-aged males, evenly distributed between the age brackets of the 30s–40s and 60s, underscoring that both younger and older individuals with HIV share a similar risk profile for ocular manifestations.

Interestingly, although our observed prevalence of ocular manifestations was 10.55%, this rate was notably lower than that reported in international studies and a previous Japanese study conducted in 1993 [4,9,10,11,12]. Factors like Japan’s national health insurance and HIV-specific financial support mechanisms, which are called “medical care for services and supports for persons with disabilities”, might explain this reduced prevalence. By shouldering most cART expenses, the Japanese government significantly promoted cART uptake, which likely aided in restoring immune states and curtailing HIV transmission. Despite this support, the cost remains high for patients with HIV infection. A standard cART can cost USD 2100 per month, and even with government assistance, it still costs USD 200 per month [13]. The AIDS Data Hub reported that by 2021, 69% of patients with HIV in Japan were receiving cART [3]. In contrast, 52.2% of the study participants received or were receiving cART when their ocular manifestations surfaced.

The ocular manifestations varied; posterior involvement was more common than anterior involvement in patients with HIV. In our study, the prevalence of uveitis has significantly burgeoned in the cART era compared to previous Japanese studies [8,12]. A report from China indicated a significant increase in the number of patients with HIV having uveitis compared to the period before 2003 (pre-HAART era) [14]. Common types of uveitis in patients with HIV infection included co-infection-related uveitis, HIV-induced uveitis, and immune recovery uveitis (IRU). In our study, three patients were diagnosed with syphilitic uveitis and one with VZV iritis. The average CD4+ T-cell count of patients with uveitis was 433.2 ± 535.6 cells/μL, implying an unsatisfactory immune status. Furthermore, we observed no discernible differences in CD4+ T-cell counts among these patients. The significantly decreased number of CD4+ T cells in patients with HIV makes them more susceptible to co-infections, including syphilis, tuberculosis (TB), and CMV. The incidence of syphilis and TB infections has increased since the early 20th century, both globally and in Japan. Given the variable and often non-specific clinical presentations of syphilitic uveitis, ophthalmologists must maintain heightened vigilance when treating patients with HIV. Prompt diagnostic testing is imperative once a patient with HIV manifests uveitis.

We also found that uveitis was the predominant ocular complication associated with HIV infection in patients who did not undergo cART. Three patients had unclassified uveitis and had not received cART when they developed uveitis. This raises the possibility that uveitis is directly induced by HIV, although further research is warranted to confirm this finding.

CMV retinitis is another common HIV-related ocular disease in patients with HIV. In our study, CMV retinitis was the second most common ocular disease, with a prevalence of 26.09%. In studies from other countries, the prevalence of CMV retinitis ranges from 2.5 to 19.7% [9,15]. Previous studies showed that cART could reduce the prevalence of CMV retinitis [9,16]. Compared to a report in 1993 [8], the incidence of CMV retinitis in patients with HIV in Japan decreased from 46% to 26% in our study. However, studies from other countries have shown that CMV retinitis remained unchanged between the pre- and post-HAART eras [14]. This may be related to the different regions and ethnicities of the participants included in the studies. In our study, more patients with CMV retinitis were receiving or had received cART compared to those who were not, consistent with findings in previous studies. According to Jabs et al. [17], a significant number of patients are diagnosed with CMV retinitis after receiving cART. In a study in Thailand, almost all newly diagnosed patients with CMV retinitis start cART before diagnosis [18]. This may relate to cART failure or indicate that these patients are in the early stages of immune reconstitution. Among those five patients who were receiving or had received cART in our study, three patients had a CD4+ T-cell count below 200 cells/μL, suggesting that the immune function of these patients has not yet been reconstituted. Therefore, regular ophthalmic examinations should be performed, even in patients with HIV who have started cART. Moreover, we continue to emphasize the significance of consistent cART in preventing and managing CMV retinitis.

CMV retinitis is also associated with CD4+ T-cell levels. However, in this study, we did not find any difference in the incidence of CMV retinitis among patients with different CD4+ T-cell levels. Di et al. also found that the occurrence of CMV retinitis is not related to CD4+ T-cell count [14]. Other studies have suggested that the prevalence of CMV retinitis remains unchanged within groups with different CD4+ T-cell counts [19]. This may be related to the time lag between the diagnosis of CMV retinitis and initiation of cART. In vitro studies have shown that controlling CMV replication requires the recovery of specific CD4+ T-cell subtypes [20,21]. Therefore, some CMV retinitis cases may have been diagnosed during the delayed reconstitution of anti-CMV immune capacity.

Our study showed that all patients with RD underwent cART. Among the four patients with RD, one had CMV retinitis, two had high myopia, and one had iritis after RD. Previous studies demonstrated that RD is an important complication of CMV retinitis. According to studies conducted in the United States, low CD4+ T-cell levels and high CMV viral loads are risk factors for RD [22]. However, the CD4+ T-cell count of patients with CMV retinitis and RD in our study was above 500 cells/μL. This observation aligns with a recent study from Thailand, which also reported no significant correlation between CD4+ T-cell levels and RD [18]. These disparities may be attributed to variations in patient demographics. High myopia is considered a risk factor for RD. Recently, the incidence of high myopia has increased annually, particularly in modern cities like Tokyo [23]. Accordingly, the number of patients with HIV who have high myopia is increasing. Currently, no studies address whether individuals with high myopia and HIV infection have an increased risk of RD, emphasizing the need for further research on this topic. Overall, RD is a major cause of visual acuity loss in patients with HIV [24]. Regular ocular examinations are invaluable for patients with HIV at an elevated risk of RD because early detection can mitigate the onset of blindness.

Our study had several limitations. First, we encountered challenges in gathering a large sample of patients with HIV from the ophthalmology outpatient unit of a tertiary hospital, underscoring the need for expansive multicenter studies in Japan. Second, a potential bias existed in our representation of ocular diseases. Patients with clearly diagnosed ocular conditions may have opted for immediate attention at primary or secondary ophthalmic clinics, potentially skewing our observations. Finally, our data collection was not exhaustive; we could not ensure comprehensive follow-up for all participants, limiting our ability to thoroughly explore patient outcomes and the various factors such as viral load affecting the visual acuity of patients with HIV.

## 5. Conclusions

In conclusion, over the past decade, significant shifts in ocular manifestations among patients with HIV infection have been observed, particularly in the context of cART availability in Japan. Our findings underscore the evolving nature of these manifestations in the cART era, with uveitis gaining prominence and the prevalence of CMV retinitis seeing a relative decrease. However, the sustained incidence of CMV retinitis in cART recipients has drawn attention to the complexities and implications of immune reconstitution. The role of factors such as high myopia in patients with HIV infection and their association with RD requires further exploration. Although the CD4+ T-cell count remains a pivotal marker in assessing immune status, its direct correlation with specific ocular manifestations, such as CMV retinitis and RD, remains inconclusive, underscoring the nuanced nature of HIV-related ocular diseases. Overall, our findings highlight the importance of regular ophthalmic examinations for early detection and intervention, emphasizing the evolving landscape of ocular complications in patients with HIV in the context of widespread cART use. These insights can inform both clinical practice and future research focusing on enhancing the quality of life of patients with HIV infection.

## Figures and Tables

**Table 1:** Demographic and laboratory characteristics of HIV-infected patients.

Parameters	Number of Patients (Percentage)
Gender	
male	23 (100%)
Age distribution (year)	Mean 53.13 ± 12.94
31–40	4 (17.4%)
41–50	4 (17.4%)
51–60	8 (34.8%)
61–70	4 (17.4%)
71–80	3 (13.0%)
Patients with cART	12 (52.2%)
Mean CD4+ T-cell count (cells/µL)	416.4 ± 475.7

cART = combination antiretroviral therapy.

**Table 2:** List of ophthalmic manifestations in HIV patients (*n* = 23).

Ocular Manifestation	Number of Patients (Percentage)	Number of Eyes (Percentage)
Total	23	36
Anterior segment	12	17
Anterior uveitis	2 (8.70%)	2 (5.56%)
VZV Iritis	1 (4.35%)	1 (2.78%)
Unclassified uveitis	1 (4.35%)	1 (2.78%)
Cataract	9 (39.13%)	14 (38.89%)
Posterior segment	20	27
Posterior/pan uveitis	12 (52.18%)	17 (47.32%)
CMV retinitis	6 (26.09%)	7 (19.44%)
Syphilitic chorioretinitis	3(13.04%)	5(13.89%)
Unclassified uveitis	3(13.04%)	5(13.89%)
Retinal detachment	4 (17.39%)	6 (16.67%)
HIV-related retinopathy	1 (4.35%)	1 (2.78%)
CRVO	1 (4.35%)	1 (2.78%)
PVR	1 (4.35%)	1 (2.78%)
Optic disc pit maculopathy	1 (4.35%)	1 (2.78%)
Adnexal	2	2
Strabismus	1 (4.35%)	1 (2.78%)
Herpes zoster ophthalmicus	1 (4.35%)	1 (2.78%)
Others	1	1
NTG	1 (4.35%)	1 (2.78%)

VZV iritis = varicella-zoster virus iritis; CMV = cytomegalovirus; PVR = proliferative vitreoretinopathy; NTG = normal-tension glaucoma; CRVO = central retinal vein occlusion.

**Table 3:** Comparison of frequency and *p* value of the CD4 counts with different ophthalmic manifestations (*n* = 18).

Ocular Manifestation	CD4 T Cells < 100	CD4 T Cells > 100	Mean CD4	*p* Value
Total	6	12		
Uveitis (overall)	4	8	433.2 ± 535.6	>0.999
VZV iritis	1	/	72	/
CMV retinitis	2	4	585.0 ± 734.3	>0.999
Syphilitic chorioretinitis	1	1	212.5 ± 163.3	>0.999
Unclassified uveitis	/	3	397.0 ± 176.3	/
Cataract	2	6	655.5 ± 610.0	0.638
Retinal detachment	/	3	585.0 ± 397.5	/
Extra-ocular manifestation	1	/	72	/

CMV = cytomegalovirus; VZV iritis = varicella-zoster virus iritis.

**Table 4:** Comparison of frequency and *p* value of cART treatment with different ophthalmic manifestations (*n* = 23).

Ocular Manifestation	Received/Receiving cART		Not Yet Received cART		*p* Value
	Number of Individuals	Number of Eyes	Number of Individuals	Number of Eyes	
Total	12	19	11	17	
Uveitis (overall)	8	11	6	8	0.0751
Unclassified uveitis	5	6	1	1	0.0217 *
Syphilitic chorioretinitis	1	2	2	3	>0.999
CMV retinitis	2	3	3	4	>0.999
Cataract	4	7	5	7	0.704
Retinal Detachment	4	6	/	/	
Strabismus	1	2	/	/	
PVR	1	1	/	/	
Retinal hemorrhage	1	1	/	/	
Pit maculopathy	1	1	/	/	
CRVO	/	/	1	1	
NTG	/	/	1	2	

CMV = cytomegalovirus; PVR = proliferative vitreoretinopathy; NTG = normal-tension glaucoma; CRVO = central retinal vein occlusion. * *p* < 0.05.

## Data Availability

All data related to this study are presented and published here.
